# Microstructure and optical properties of Pr^3+^-doped hafnium silicate films

**DOI:** 10.1186/1556-276X-8-43

**Published:** 2013-01-21

**Authors:** YongTao An, Christophe Labbé, Larysa Khomenkova, Magali Morales, Xavier Portier, Fabrice Gourbilleau

**Affiliations:** 1 CIMAP, UMR 6252 CNRS/CEA/Ensicaen/UCBN, 6 Boulevard Maréchal Juin, Caen, Cedex 4, 14050, France

**Keywords:** Praseodymium, Hafnium silicate, Oxygen vacancies, Photoluminescence, Energy transfer

## Abstract

In this study, we report on the evolution of the microstructure and photoluminescence properties of Pr^3+^-doped hafnium silicate thin films as a function of annealing temperature (*T*_A_). The composition and microstructure of the films were characterized by means of Rutherford backscattering spectrometry, spectroscopic ellipsometry, Fourier transform infrared absorption, and X-ray diffraction, while the emission properties have been studied by means of photoluminescence (PL) and PL excitation (PLE) spectroscopies. It was observed that a post-annealing treatment favors the phase separation in hafnium silicate matrix being more evident at 950°C. The HfO_2_ phase demonstrates a pronounced crystallization in tetragonal phase upon 950°C annealing. Pr^3+^ emission appeared at *T*_A_ = 950°C, and the highest efficiency of Pr^3+^ ion emission was detected upon a thermal treatment at 1,000°C. Analysis of the PLE spectra reveals an efficient energy transfer from matrix defects towards Pr^3+^ ions. It is considered that oxygen vacancies act as effective Pr^3+^ sensitizer. Finally, a PL study of undoped HfO_2_ and HfSiO_*x*_ matrices is performed to evidence the energy transfer.

## Background

Rare-earth elements are important optical activators for luminescent devices. Among various rare-earth luminescent centers, trivalent praseodymium (Pr^3+^) offers simultaneously a strong emission in the blue, green, orange, and red spectral range, satisfying the complementary color relationship [1,2]. Consequently, Pr^3+^-doped glass/crystals are often used as phosphor materials [2,3]. SiO_2_-(Ca, Zn)TiO_3_:Pr^3+^ phosphors prepared with nanosized silica particles exhibit an intense red photoluminescence (PL) [3]. The Pr^3+^ emission was achieved for Si-rich SiO_2_ (SRSO) implanted with Pr^3+^ ions, but its intensity was lower [4].

Hafnium dioxide (HfO_2_) and hafnium silicates (HfSiO_*x*_) are currently considered as the predominant high-*k* dielectric candidates to replace the conventional SiO_2_ due to the rapid downscaling of the complementary metal-oxide semiconductor (CMOS) transistors [5,6]. It is ascribable to their good thermal stability in contact with Si, large electronic bandgaps, reasonable conduction band offset in regard to Si, and their compatibility with the current CMOS technology. Our group has first explored the structural and thermal stability of HfO_2_-based layers fabricated by radio frequency (RF) magnetron sputtering [7,8] and their nonvolatile memory application [9,10].

It is worth to note that both HfO_2_ and HfSiO_*x*_ matrices have lower phonon frequencies compared to those of SiO_2_, and as a consequence, both are expected to be suitable hosts for rare-earth activators. Thus, PL properties have been investigated for the HfO_2_ matrix doped with Tb^3+^[11], Eu^3+^[11,12], or Er^3+^[12,13] and have been explained by the interaction of rare-earth ions with host defects. Recently, our group has demonstrated that an enhancement of Er^3+^ PL emission can be achieved for the Er-doped HfSiO_*x*_ matrix in comparison with that of the Er-doped HfO_2_[14]. It was also observed that an energy transfer from the HfO_2_ host defects towards Er^3+^ ions, whereas the existence of Si clusters allowed an enhancement of the Er^3+^ ion emission under longer-wavelength excitation. Consequently, the mechanism of the excitation process, when Si clusters and oxygen-deficient centers act as Er^3+^ sensitizers, has been proposed to explain an efficient rare-earth emission from Er-doped HfSiO_*x*_ hosts [14] similar to that observed for the Er-doped SRSO materials [15].

In this paper, we study the microstructure and optical properties of Pr-doped hafnium silicate films fabricated by magnetron sputtering versus annealing temperature. We demonstrate that an efficient Pr^3+^ light emission is achievable by tuning the annealing conditions. The excitation mechanism of Pr^3+^ ions is also discussed.

## Methods

The films were deposited onto p-type (100) 250-μm-thick Si wafers by RF magnetron sputtering of a pure HfO_2_ target topped by calibrated Si and Pr_6_O_11_ chips. The growth was performed in pure argon plasma with an RF power density of 0.98 W∙cm^−2^; the Si substrate temperature was kept at 25°C. After deposition, a post-annealing treatment was carried out under a nitrogen flow, at temperatures (*T*_A_) varying from 800°C up to 1,100°C for 1 h.

The refractive index (*n*) (given always at 1.95 eV) and the film thicknesses were deduced from spectroscopic ellipsometry data. The chemical composition of the films was determined by Rutherford backscattering spectrometry (RBS) using a 1.5-MeV ^4^He^+^ ion beam with a normal incidence and a scattering angle of 165°. The infrared absorption properties were investigated by means of a Nicolet Nexus (Thermo Fisher Scientific, Waltham, MA, USA) Fourier transform infrared (FTIR) spectroscopy at Brewster’s incidence (65°) in the range of 500 to 4,000 cm^−1^. X-ray diffraction (XRD) experiments were performed using a Philips Xpert MPD Pro device (PANalytical B.V., Almelo, The Netherlands) with CuK*α* radiation (*λ* = 1.5418 Å) at a fixed grazing angle incidence of 0.5°. Cross-sectional specimens were prepared by standard procedure involving grinding, dimpling, and Ar^+^ ion beam thinning until electron transparency for their observation by transmission electron microscopy (TEM). The samples were observed using a FEG 2010 JEOL instrument, operated at 200 kV. The PL emission and PL excitation (PLE) measurements were carried out using a 450-W Xenon arc lamp as excitation source at room temperature corrected on spectral response with the help of a Jobin-Yvon Fluorolog spectrometer (HORIBA Jobin Yvon Inc., Edison, NJ, USA).

## Results and discussion

### Composition and structural characterizations

**Figure 1:**
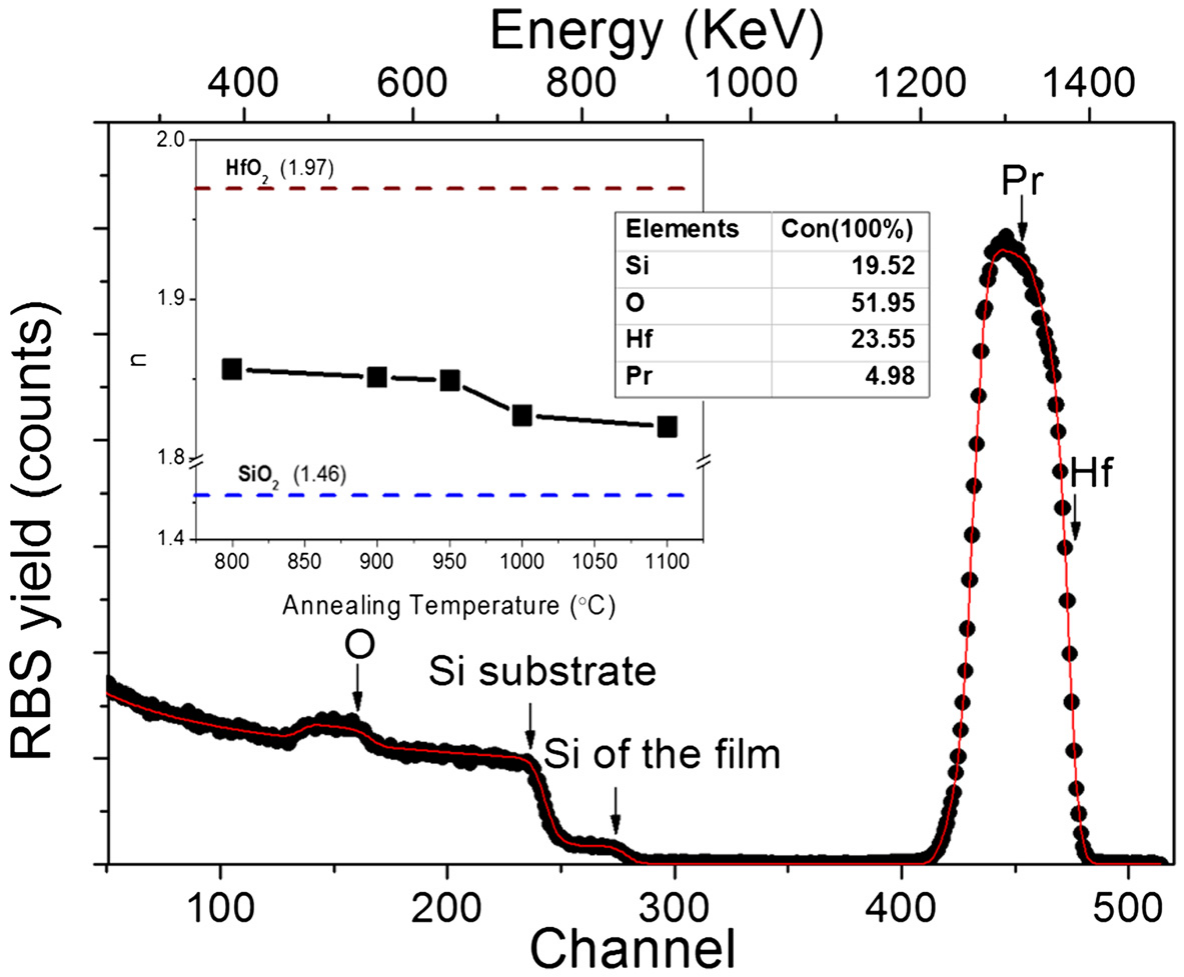
**Experimental RBS spectrum (points) and simulated curve using SIMNRA (solid line) for as-deposited film.** Inset table is the chemical composition of the film. Inset figure is the refractive index evolution versus *T*_A_. The pure HfO_2_ and pure SiO_2_ indices are also shown in dashed lines. The films are about 170 nm in thickness.

In this study, the chemical composition of the film Hf_0.24_Si_0.20_O_0.52_Pr_0.05_ was determined through the simulation of the corresponding RBS spectrum using the SIMNRA program (Figure 1). The RBS analysis shows that the as-deposited film cannot be considered as a matrix of SiO_2_ and HfO_2_ only, as this is usually assumed for hafnium silicates. In our case, we deal with a hafnium silicate matrix enriched with silicon as well as doped with Pr^3+^ ions.

The inset of Figure 1 displays the refractive index evolution upon annealing treatment between 800°C and 1,100°C. The uncertainty of the refractive index is 0.01. Nevertheless, it was notable that it decreased with *T*_A_. In a previous study on as-deposited film, it was found that the refractive index was about 2.2 [8], exceeding the value corresponding to the stoichiometric HfSiO_4_ matrix (1.7) due to Si enrichment [8]. However, upon annealing, the refractive index is found to be about 1.85 (*T*_A_ = 800°C) and 1.82 (*T*_A_ = 1,100°C). If we exclude the decrease of porosity, this evolution could be explained by the increasing contribution of some phases with lower refractive index upon annealing (like SiO_2_ (1.46)) [8].

**Figure 2:**
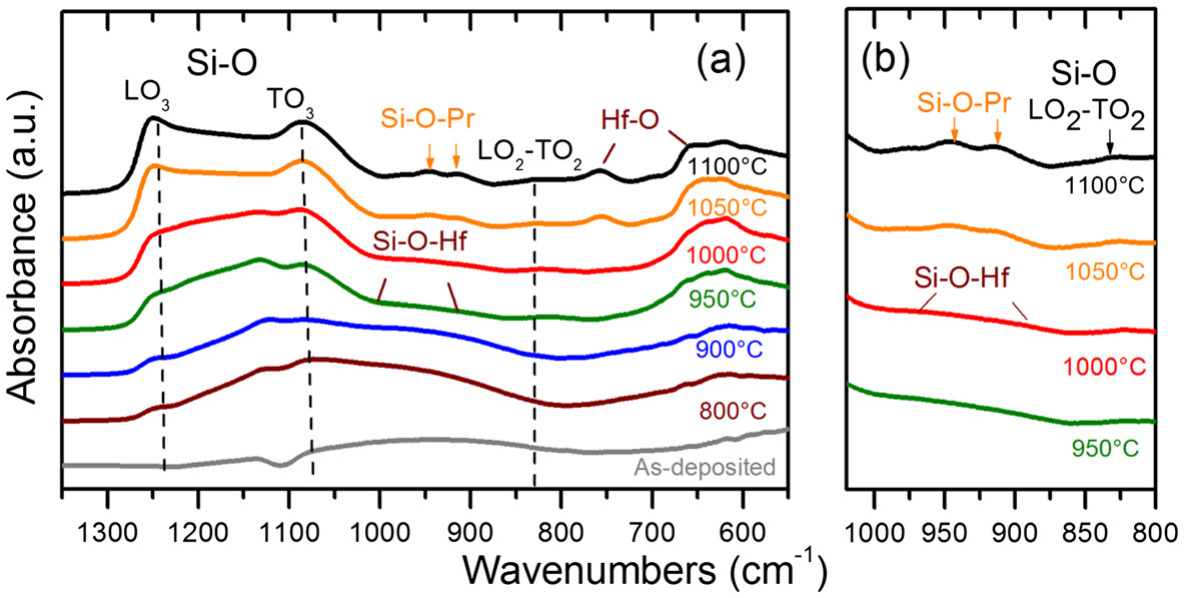
**FTIR spectra of samples and detailed spectra between 800 and 1,020 cm^−1^.** (**a**) FTIR spectra of samples measured at Brewster’s angle (65°) as a function of *T*_A_ for 1 h of nitrogen flow. Si-O bands are marked by dashed lines. (**b**) Detailed spectra between 800 and 1,020 cm^−1^ for better observation of the peak position in this range.

Figure 2a represents the evolution of the FTIR spectra as a function of *T*_A_. The FTIR spectrum of as-deposited film is represented by two broad vibration bands in the ranges of 500 to 750 and 800 to 1,200 cm^−1^. An annealing treatment stimulates the appearance of several bands that peaked at about 827, 1,084, and 1,250 cm^−1^ (dashed lines in Figure 2a) corresponding to the LO_2_-TO_2_, TO_3_, and LO_3_ vibration modes of the Si-O bond, respectively. Moreover, the increase of the LO_3_ mode intensity is attributed to the increase in the number of Si-O-Si bonds. This is a signature of the formation of the SiO_2_ phase due to a phase separation process, leading to the decrease of the refractive index for *T*_A_ ≥ 800°C.

This phase separation is confirmed also by an increase of the vibration mode intensity in the range of 600 to 780 cm^−1^, corresponding to Hf-O bonds for the formation of the HfO_2_ phase [7,14]. The appearance of well-defined peaks at 760 and 660 cm^−1^ for *T*_A_ ≥ 1,050°C attests the presence of the monoclinic HfO_2_ phase [16]. Besides, for *T*_A_ ≥ 1,050°C, two new absorption peaks that centered at 900 and 1,000 cm^−1^ appeared (detailed in Figure 2b). As we showed earlier [8], at such temperature, undoped HfSiO_*x*_ did not reveal the presence of Si-O-Hf bonds. Thus, the vibration band at 900 and 1,000 cm^−1^ can be attributed to Si-O-Pr asymmetric mode. Similar incorporation of rare-earth ions into Si-O bonds and the formation of rare-earth silicate phase was observed earlier for SiO_*x*_ materials doped with Er^3+^, Nd^3+^, or Pr^3+^and annealed at 1,100°C [17-19]. Thus, based on this comparison, one can conclude about the formation of Pr silicate revealed by FTIR spectra.

**Figure 3:**
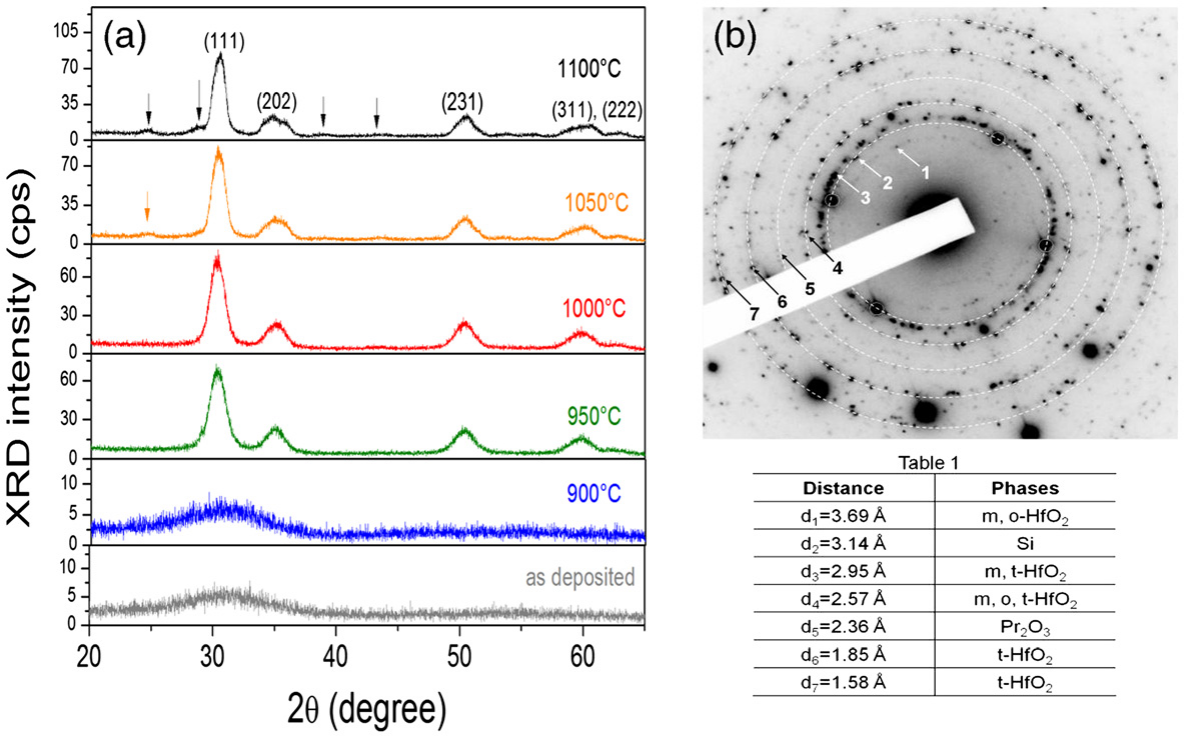
**XRD and SAED patterns.** (**a**) XRD patterns of as-deposited and annealed films. (**b**) SAED pattern of the 1,100°C-annealed film. Table one is the *d* spacing list obtained from (b) and the corresponding phases.

To get more information about the evolution of film structure, we performed XRD analyses. For as-deposited and 900°C annealed films, XRD spectra show a broad peak in the range of 25.0° to 35.0° with a maximum intensity located at 2*θ* ≈ 31.0° (Figure 3a). The shape of the XRD peak demonstrates the amorphous nature of both layers. With *T*_A_ increase, several defined peaks appear, emphasizing the formation of a crystalline structure. Thus, for *T*_A_ = 950°C, intense XRD peaks at 2*θ* ≈ 30*.*3°, 35*.*0°, and 50*.*2° were detected. They correspond to the (111), (200), and (220) planes of the tetragonal HfO_2_ phase, respectively, confirming the FTIR analysis [8]. The peak at 2*θ* ≈ 60.0° can be considered as an overlapping of the reflections from the (311) and (222) planes of the same HfO_2_ phase. When *T*_A_ reaches 1,050°C, the appearance of peaks at almost 2*θ* ≈ 24.6° and 28.5° occurs. The first peak is attributed to the monoclinic HfO_2_ phase (Joint Committee on Powder Diffraction Standards (JCPDS) no. 78–0050). The second one, at 28.5°, could be ascribed to several phases such as Pr_2_O_3_ (2*θ*_[222]_ ≈ 27.699°) (JCPDS no. 78–0309), Pr_6_O_11_ (2*θ*_[111]_ ≈ 28.26°) (JCPDS no. 42–1121), Si (2*θ*_[111]_ ≈ 28.44°) (JCPDS no. 89–5012), or Pr_2_Si_2_O_7_ (2*θ*_[008]_ ≈ 29.0°) (JCPDS no. 73–1154), due to the overlapping of corresponding XRD peaks. This observation is in agreement with the FTIR spectra (Figure 2b) showing the Hf-O vibrations and formation of Pr clusters.

In some oxygen-deficient oxide films [20,21], the phase separation is observed with the crystallization of the stoichiometric oxide matrix in the initial step and then in metallic nanoclustering. The aforesaid results are also coherent with our previous study of nonstoichiometric Hf-silicate materials in which we have evidenced the formation of HfO_2_ and SiO_2_ phases as well as Si nanoclusters (Si-ncs) upon annealing treatment [14,22]. To underline this point, we performed a TEM observation of 1,100°C annealed sample and observed a formation of crystallized Si clusters. Figure 3b exhibits the corresponding selected area electron-diffraction (SAED) pattern. The analysis of dotted diffraction rings indicates the presence of several phases. Among them, one can see the signature of monoclinic and tetragonal HfO_2_ phases, Pr_2_O_3_ phase, and crystallized Si phase (the Table one found in Figure 3b). This latter confirms the presence of Si-ncs but in a small amount (a few spots on the corresponding ring).

### Photoluminescence properties

**Figure 4:**
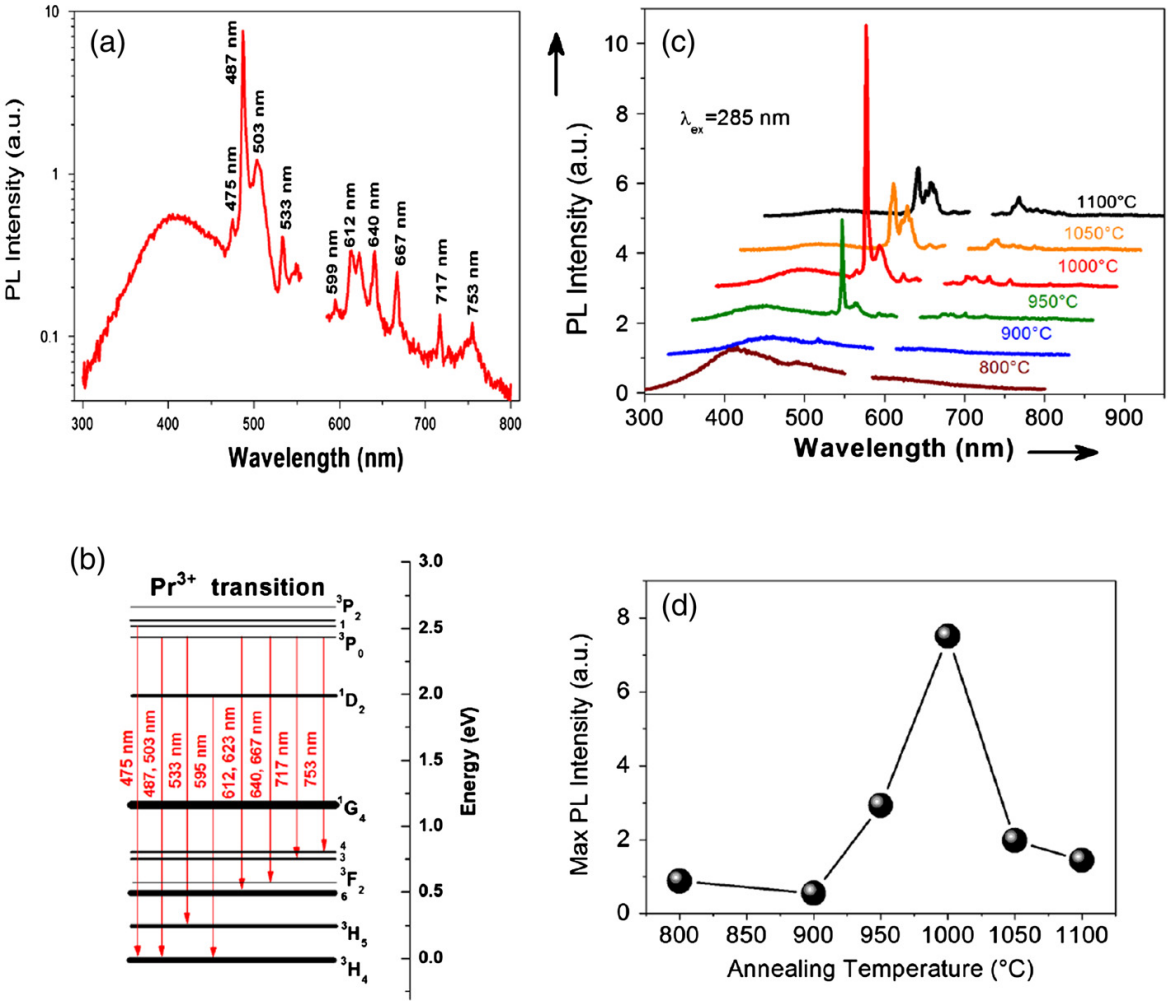
**PL spectra, schematics, and PL behavior.** (**a**) PL spectrum in logarithmic scale for 1,000°C annealed layer. (**b**) The schematics of the Pr^3+^ intra-4f transitions. (**c**) PL spectra of the films annealed at different annealing temperatures (*T*_A_= 800°C to 1,100°C). The excitation wavelength is 285 nm. (**d**) The behavior of the PL intensity of the ^3^P_0_→^3^H_4_ transition at 487 nm with *T*_A_.

Figure 4a shows the PL spectra of Pr^3+^-doped hafnium silicate films, which were excited by a 285-nm wavelength for Pr^3+^ ions. Remarkable emission is observed with peaks centered at about 475, 487, 503, 533, 595, 612, 623, 640, 667, 717, and 753 nm. They are associated to the Pr^3+^ energy level transitions ^3^P_1_→^3^H_4_, ^3^P_0_→^3^H_4_, ^3^P_0_→^3^H_5_, ^1^D_2_→^3^H_4_, ^3^P_0_→^3^H_6_, ^3^P_0_→^3^F_2_, ^3^P_0_→^3^F_3_, and ^3^P_0_→^3^F_4_, respectively, as shown in Figure 4b [23]. The maximum emission intensity corresponds to the peak centered at 487 nm due to the ^3^P_0_→^3^H_4_ transition.

On the first step, the effect of annealing on Pr^3+^ PL properties was investigated (Figure 4c). The PL intensity evolution is shown in Figure 4d for the representative peak at 487 nm. The PL intensity increases with *T*_A_ rising from 800°C up to 1,000°C and then decreases with further *T*_A_ increase. At the initial stage, the annealing process is supposed to decrease the non-radiative recombination rates [24]. Thereafter, the quenching of the Pr^3+^ emission that occurred for *T*_A_ > 1,000°C can be due to the formation of the Pr^3+^ silicate or Pr oxide clusters (Figure 2) similar to the case observed in [17,18]. Moreover, it is interesting to note that the position of peak (Pr^3+^: ^3^P_0_→^3^H_4_) redshifts from 487 nm (*T*_A_ ≤ 1,000°C) to 492 nm (*T*_A_ = 1,100°C) as shown in Figure 4c. At the same time, two split peaks contributed to the ^1^D_2_→^3^H_4_ transition that joined as one sharp peak which centered at 617 nm. All these results can be explained by the dependence of Pr^3+^ PL parameters on the crystal field associated with the type of Pr^3+^ environment [25]. Furthermore, the Pr^3+^ surrounding has been influenced by the crystallization of the HfO_2_ phase for films annealed at *T*_A_ > 1,000°C.

Taking into account the formation of Si-ncs in Pr-doped HfSiO_*x*_ samples annealed at 1,100°C for 1 h, one can expect the appearance of a PL emission due to exciton recombination inside Si-ncs, which is usually observed in the 700- to 950-nm spectral range [17,18]. However, our study of these samples did not reveal the Si-nc PL emission. Two reasons can be mentioned. The first one is the low density of Si-ncs, confirmed by the SAED pattern (Figure 3b). The second one is the efficient energy transfer from the Si-ncs to Pr^3+^ ions. However, based on the comparison of energetic diagrams of Pr^3+^ ions and Si-ncs, we observed that the energy levels of Si-ncs and Pr^3+^ ions have no overlapping. Thus, the energy transfer from Si-ncs toward Pr^3+^ ions should be very weak, contrary to an efficient sensitizing of other rare-earth ions such as Er^3+^ or Nd^3+^ in SiO_*x*_ or HfSiO_*x*_ matrices [23,24]. Thus, in the case of Pr-doped HfSiO_*x*_ samples, Si-ncs do not seem to be a major actor for the energy transfer. Nevertheless, due to the low amount of Si-ncs, their PL signal is not detectable.

Thus, the second step of our investigation was to study the mechanism of Pr^3+^ energy transfer under the 285-nm excitation wavelength. The energy diagram of Pr^3+^ ions does not present such an absorption band wavelength at 285 nm (Figure 4b). In addition, the 4*f* to 5*d* transition is witted in upper energy level between 250 and 220 nm [26]. This evidences the indirect excitation of Pr^3+^ ions by the 285-nm wavelength and confirms an energy transfer behavior. To investigate this behavior in detail, we take interest in the strong background PL from 350 to 550 nm for the layers annealed at 800°C to 900°C in Figure 4c. This broad band may be ascribed to more than one kind of defect [5,6,27]. For the layers annealed at higher *T*_A_ such as 1,000°C, the intensity of this PL band drops deeply while the Pr^3+^ PL intensity increases notably. This suggests that the energy transfers from host defects to Pr^3+^ ions.

**Figure 5:**
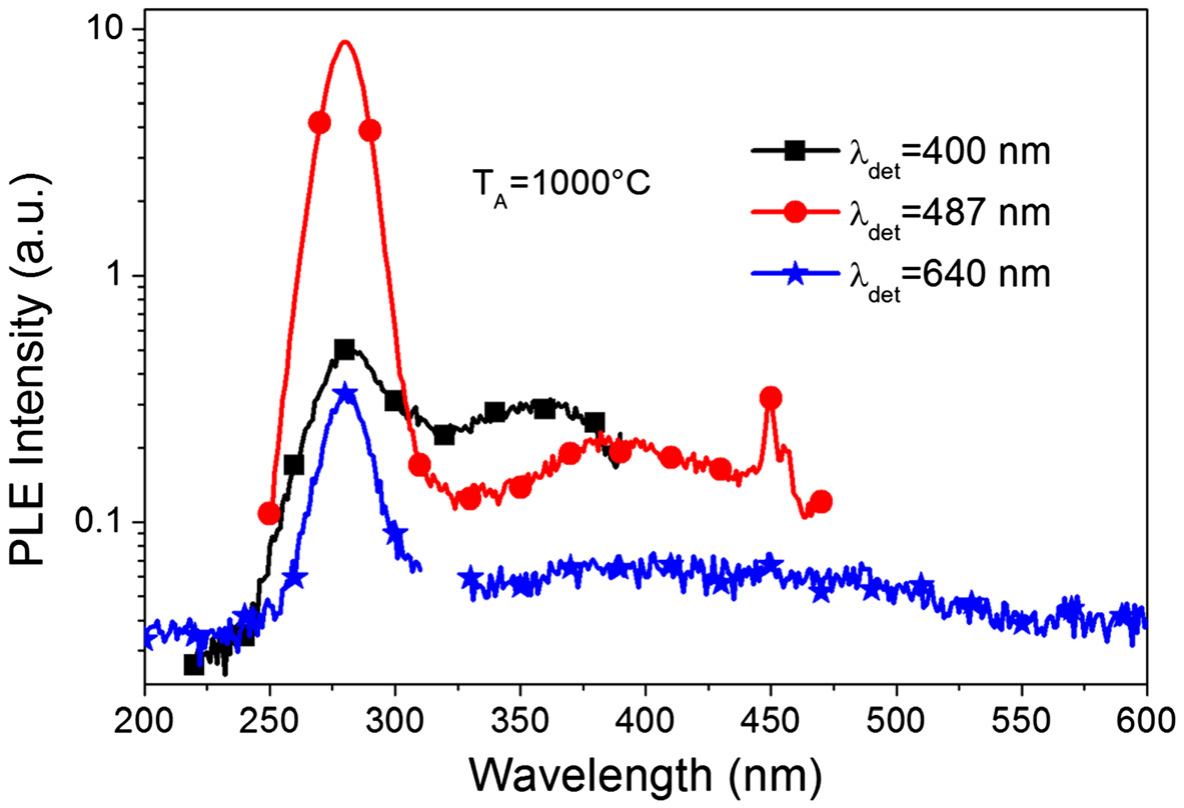
PLE spectra in logarithmic scale for 1,000°C annealed layer detected for different emission peaks.

To understand this point, PLE spectra were recorded for the ‘optimized’ sample (annealed at 1,000°C) at different detection wavelengths (400, 487, and 640 nm, corresponding almost to the background emission for the former and to Pr^3+^ PL for the two latter), and they are presented in Figure 5. All the PLE spectra show a remarkable peak at about 280 nm (4.43 eV), and this peak position is in good agreement with that observed for oxygen vacancies [28]. According to some references [6,29], the O vacancies in the host matrix introduce a series of defect states (at about 1.85 to 4.45 eV) in the bandgap of HfO_2_, which might provide recombination centers for excited *e* and *h* pairs. These excitons can effectively transfer energy to the nearby Pr^3+^ ions due to the overlapping with absorption levels of Pr^3+^ and, thus, to enhance the Pr^3+^ PL emission. Therefore, the Hf-related O vacancies in the host matrix serve as effective sensitizers to the adjacent Pr ions. An additional argument for this interaction is the increasing of Pr^3+^ PL intensity with *T*_A_ (from 900°C to 1,000°C) which caused the formation of HfO_2_ grains, providing more Hf-related O vacancies. However, due to a decomposition process, formation of the Si-rich phase (Pr-doped SiO_*x*_ and/or Pr silicate) occurs too. The decrease of the intensity of the PL band that peaked at 400 nm and the increase of corresponding Pr^3+^ emission are a signature of the contribution of these Si-rich phase to the Pr^3+^ ion excitation (Figure 4c).

**Figure 6:**
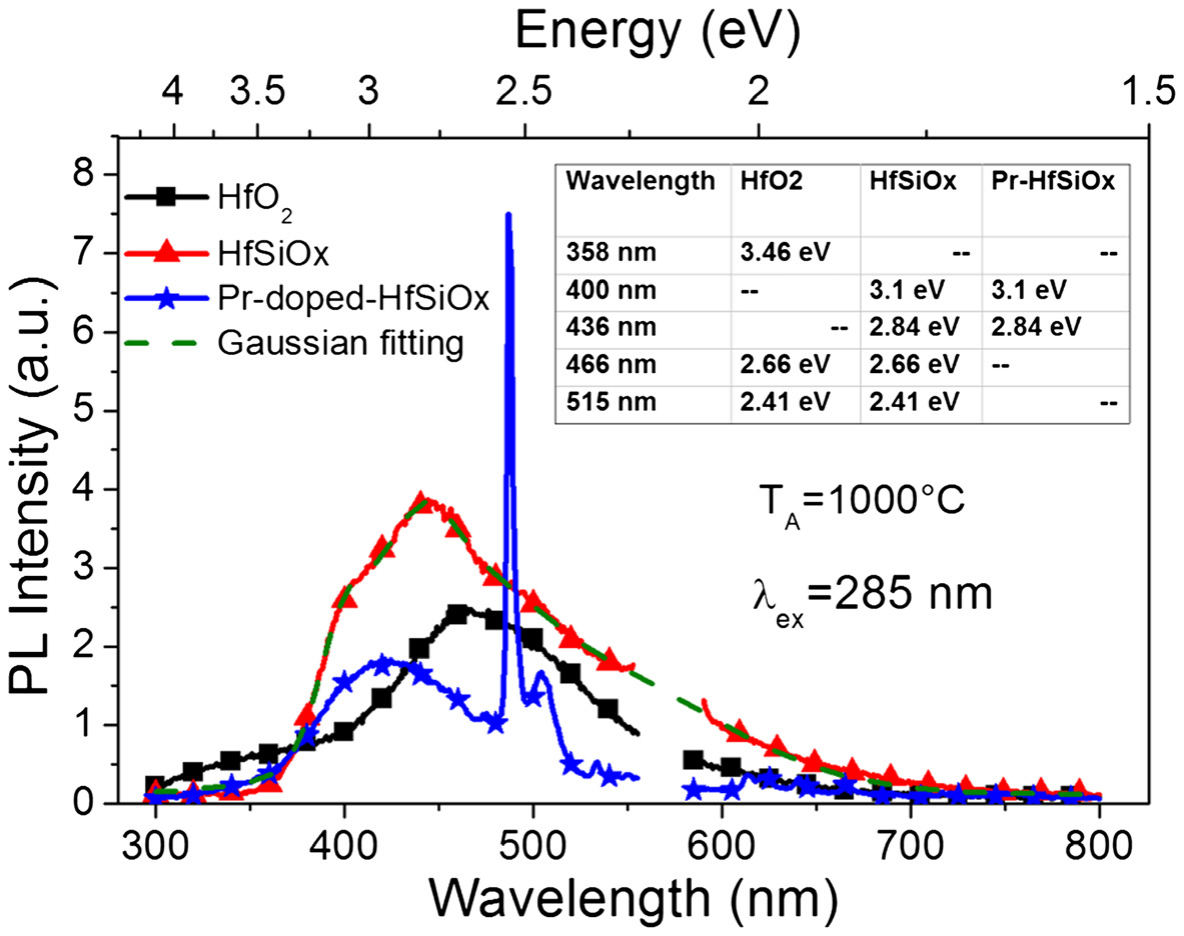
**PL spectra of Pr-doped and undoped HfSiO**_***x***_**and undoped pure HfO**_**2**_**films excited at 285 nm.** The films were annealed at 1,000°C. Inset table is data of the fitting peaks.

The excitation mechanism of Pr^3+^ ions was further explored by comparing two matrices. We carried out the PL experiments for three kinds of samples annealed at 1,000°C: undoped HfO_2_, undoped HfSiO_*x*_ films, and Pr-doped HfSiO_*x*_ films excited by a 285-nm source (Figure 6). According to [6], in HfSiO_*x*_ films, two types of O vacancies coexist: one is an O vacancy surrounded by Si atoms (Si-related O vacancy), while the other is an O vacancy surrounded by Hf atoms (Hf-related). Since the HfO_2_ phase is ionic, it is obvious that it forms easier in the HfSiO_*x*_ film upon annealing, and thus, Hf-related O vacancy formation is most preferable than Si-related O vacancy [6]. Herein, a particular interest is focused on the emissions from the defects: the Pr-doped film shows a broad band peaked at 420 nm, while the peak positions redshift to about 450 and 490 nm for HfSiO_*x*_ and HfO_2_ films, respectively. The 450-nm band can be fitted in energy into four Gaussian bands centered at 3.1, 2.84, 2.66, and 2.11 eV (table inset of Figure 6). The former two peaks are related to defects of the SiO_*x*_ phase, for instance, Si-related oxygen deficient centers [13,28]. The peak at 2.66 eV is ascribed to O vacancies related to the HfO_2_ phase. The disappearance of the 2.66-eV PL component is accompanied with the appearance of the strong 487-nm emission and series of other Pr^3+^ transitions in Pr-doped HfSiO_*x*_ film, which implies the energy transfer from O vacancies to the Pr sites.

As a result, the Si-rich HfO_2_ host not only serves as a suitable matrix to achieve efficient Pr^3+^ emission, but also provides a sufficient amount of O vacancies acting as effective sensitizers of rare-earth ions.

## Conclusions

In summary, we have fabricated the Pr^3+^-doped hafnium silicate layers by RF magnetron sputtering. The effect of the annealing temperature on the film properties has been investigated by means of ellipsometry, XRD, and FTIR spectroscopies. We showed that the highest Pr^3+^ PL intensity is obtained for 1,000°C annealing. The PL and PLE measurements demonstrate that the Pr^3+^ ions were efficiently excited by oxygen vacancies in the films, and thus, remarkable Pr^3+^ PL can be obtained by a non-resonant excitation process. The present results show the promising application of Pr-doped films for future optoelectronic devices.

## Competing interests

The authors declare that they have no competing interests.

## Authors’ contributions

YTA fabricated the Pr-doped layers, carried out the characterization studies, as well as wrote the draft of manuscript. LK fabricated the undoped layers. MM performed the RBS measurements and refinements. XP performed the TEM study. CL and FG coordinated the study. All authors discussed and commented on the manuscript. All authors read and approved the final manuscript.
